# Effects of tofacitinib on lymphocyte sub-populations, CMV and EBV viral load in patients with plaque psoriasis

**DOI:** 10.1186/s12895-015-0025-y

**Published:** 2015-05-08

**Authors:** Fernando Valenzuela, Kim A Papp, David Pariser, Stephen K Tyring, Robert Wolk, Marjorie Buonanno, Jeff Wang, Huaming Tan, Hernan Valdez

**Affiliations:** Department of Dermatology, Faculty of Medicine, University of Chile and Probity Medical Research, Santiago, Chile; Clinical Research and Probity Medical Research, Waterloo, ON Canada; Department of Dermatology, Eastern Virginia Medical School and Virginia Clinical Research Inc., Norfolk, VA USA; Department of Dermatology, University of Texas Medical School, Houston, TX USA; Pfizer Inc, Groton, CT USA; Quintiles, Cambridge, MA USA; Pfizer Inc, New York, NY USA; Present address: Statistical Consulting & Solutions, LLC, Brookline, MA USA; Specialty Care Medicines Development Group, Pfizer Inc, 219 E 42nd Street, 7th Floor Room 50, NYO 219/07/01, New York, NY 10017 USA

**Keywords:** Cytomegalovirus, Epstein-Barr virus, Janus kinase inhibition, Lymphocyte, Plaque psoriasis, Tofacitinib, Viral reactivation

## Abstract

**Background:**

Plaque psoriasis is a debilitating skin condition that affects approximately 2% of the adult population and for which there is currently no cure. Tofacitinib is an oral Janus kinase inhibitor that is being investigated for psoriasis.

**Methods:**

The design of this study has been reported previously (NCT00678210). Patients with moderate to severe chronic plaque psoriasis received tofacitinib (2 mg, 5 mg, or 15 mg) or placebo, twice daily, for 12 weeks. Lymphocyte sub-populations, cytomegalovirus (CMV) and Epstein-Barr virus (EBV) DNA were measured at baseline and up to Week 12.

**Results:**

Tofacitinib was associated with modest, dose-dependent percentage increases from baseline in median B cell count at Week 4 (24–68%) and Week 12 (18–43%) and percentage reductions from baseline in median natural killer cell count at Week 4 (11–40%). The proportion of patients with detectable CMV and EBV DNA (defined as >0 copies/500 ng total DNA) increased post-baseline in tofacitinib-treated patients. However, multivariate analyses found no relationship between changes in CMV or EBV viral load and changes in lymphocyte sub-populations or tofacitinib treatment.

**Conclusions:**

Twelve weeks of treatment with tofacitinib had no clinically significant effects on CMV or EBV viral load, suggesting that lymphocyte sub-populations critical to the response to chronic viral infections and viral reactivation were not significantly affected. Replication of these findings during long-term use of tofacitinib will allow confirmation of this observation.

## Background

Psoriasis is a debilitating and recurring immune-mediated inflammatory disease that affects 0.91–8.5% of the adult population according to geographic region [1]. Plaque psoriasis is by far the most prevalent form, accounting for approximately 80% of cases [2]. Tofacitinib is an oral Janus kinase (JAK) inhibitor that is being investigated for psoriasis. Oral tofacitinib has been evaluated in a Phase I study in patients with active psoriasis [3], and in Phase II and Phase III studies in patients with chronic plaque psoriasis [4-6] (see also NCT01276639 and NCT01309737). Tofacitinib binds to and inhibits JAK1 and JAK3, thereby blocking pro-inflammatory cytokine signaling, in particular interleukin (IL)-6 and interferon γ [7,8]. C-reactive protein (CRP), a marker of inflammation, is under the transcriptional control of IL-6 [9] and, thus, may be modulated by tofacitinib. Tofacitinib also modulates the activity of several other interleukins (including IL-2, −4, −7, −9, −15, and −21) that have roles in mediating immune response to viral infection/reactivation, lymphocyte development, and effector function [10,11]. Owing to the chronic nature of psoriasis and consequent prolonged and repeated periods of exposure to systemic immunomodulatory therapies, it is important to evaluate the potential effects of new therapies, such as tofacitinib, on immunosurveillance.

This exploratory analysis investigated the effect of oral tofacitinib on lymphocyte sub-populations to identify any impact of tofacitinib on immunosurveillance during a Phase IIb, 12-week, placebo-controlled study in patients with moderate to severe plaque psoriasis [4]. The potential for reactivation of latent viruses (eg cytomegalovirus [CMV], John Cunningham virus, Epstein-Barr virus [EBV], hepatitis B virus, and varicella zoster virus) has been described with other immunosuppressive agents [12-17]. Recurrence of CMV infection in immunocompromised patients can cause damage to the digestive system, lungs, and eyes [18]. CMV infections have been reported in patients with psoriasis receiving biologic therapies including the tumor necrosis factor inhibitor (TNFi) etanercept, and efalizumab, an anti-CD11a antibody [12,13]. Efalizumab was found to be associated with increased risk of John Cunningham virus -related progressive multifocal leukoencephalopathy, a potentially fatal infection of the central nervous system and, consequently, was withdrawn from the market in 2009 [19]. Reactivation of EBV is associated with development of lymphoproliferative malignancies including non-Hodgkin lymphoma, and has been reported in patients receiving biologic and nonbiologic treatment for psoriasis [14,15]. Reactivation of hepatitis B virus can lead to severe hepatitis, liver failure, and death [20]. A need for hepatitis B virus screening and pre-emptive therapy has been highlighted in patients receiving TNFi therapy [21]. Reactivation of latent varicella zoster virus causes shingles, the main complication of which is chronic pain, and is a long-term challenge in patients receiving immunosuppressive therapy [17].

In the present study, potential for viral reactivation in general was evaluated using CMV and EBV DNA copy numbers as surrogates of viral-specific immune surveillance. To complement evaluation of lymphocyte sub-populations during tofacitinib treatment, changes in inflammatory activity during tofacitinib treatment were assessed using serum levels of CRP as a marker.

## Methods

This was a Phase IIb, randomized, double-blind, parallel-group, placebo-controlled study conducted in 42 centers in the United States and Canada (NCT00678210). Patients were aged ≥18 years with moderate to severe chronic plaque-type psoriasis covering ≥15% of the total body surface area, had stable disease for ≥6 months, a Psoriasis Area and Severity Index (PASI) score ≥13 and were eligible for phototherapy or systemic treatment of psoriasis [4]. Patients were excluded if they had received any prior lymphocyte-depleting therapy (eg alemtuzumab, cyclophosphamide, chlorambucil) or had received rituximab/other selective B lymphocyte-depleting therapy within the preceding 12 months.

The study design has been reported elsewhere [4]. Briefly, patients were randomized 1:1:1:1 to receive oral tofacitinib (2 mg, 5 mg, or 15 mg twice daily [BID]), or placebo, for 12 weeks. Follow-up continued for 4 weeks after study completion or withdrawal. The safety population (all patients who received at least one dose of tofacitinib or placebo) was used in this exploratory analysis. The study was performed in compliance with the International Conference on Harmonization Good Clinical Practice Guidelines. All patients provided written informed consent, and both consent documentation and the final protocol were reviewed and approved by the institutional review board and/or the independent ethics committee at each of the investigational centers participating in the study.

### Cell and viral quantification

Whole blood and serum were collected and analyzed. Lymphocyte populations were identified and quantified by fluorescence-activated cell sorting (FACS) analysis at baseline, Weeks 4 and 12, or early termination. Lymphocyte subset markers analyzed included: CD3^+^ (total T cells), CD3^+^/CD4^+^ (T helper cells; T_H_), CD3^+^/CD8^+^ (cytotoxic T cells; T_C_), CD19^+^ (B cells), and CD16^+^/CD56^+^ (natural killer cells; NK).

Serum CRP levels were measured at baseline and Week 12 or early termination. Blood CMV and EBV DNA was measured at baseline, Weeks 4 and 8 (CMV only), and Week 12 or early termination. Viral loads were quantified by real-time polymerase chain reaction (PCR) using DNA isolated directly from peripheral blood leukocytes and expressed as viral DNA copies/500 ng of total DNA. CMV or EBV DNA levels >0 copies/500 ng total DNA were defined as detectable.

### Statistical analysis

For continuous endpoints measured longitudinally, a repeated mixed-effect model was used to analyze change from baseline, where treatment, visit week, and interaction between treatment and visit week were included as fixed factors, along with baseline value as the covariate. For continuous endpoints evaluated at a single time point, analysis of covariance (ANCOVA) was used to analyze change from baseline, and the baseline value was included as covariate. For categorical data, comparisons between groups were performed using a Chi-squared test. Missing values were not imputed in any of these analyses.

Spearman’s correlation analyses, based on the ranks of variables, were performed between change in viral load or CRP level, and change in lymphocyte sub-population cell counts. A linear model was used to assess potential relationships between change in viral load or CRP level, and change in lymphocyte sub-population cell counts, and doses of tofacitinib.

## Results

Baseline demographics, disease characteristics, and laboratory values (including lymphocyte sub-population cell counts, CRP, CMV, and EBV viral load) were similar between groups (Table 1).

**Table 1:** **Patient demographics, disease characteristics, cell counts, CMV and EBV DNA count at baseline**

	**Placebo (n = 50)**	**Tofacitinib 2 mg BID (n = 49)**	**Tofacitinib 5 mg BID (n = 49)**	**Tofacitinib 15 mg BID (n = 49)**
Mean age, years (SD)	43.9 (13.0)	45.7 (13.8)	44.0 (12.6)	43.6 (15.6)
Male, n (%)	36 (72.0)	29 (59.2)	29 (59.2)	31 (63.2)
White, n (%)	41 (82.0)	36 (73.5)	42 (85.7)	40 (81.6)
Mean weight, kg (SD)	89.6 (23.9)	89.6 (23.0)	92.2 (23.5)	93.1 (29.7)
Mean PASI score (SD)	21.5 (7.1)	21.5 (6.7)	21.2 (8.1)	22.6 (10.3)
Mean BSA, % (SD)	29.8 (13.5)	29.8 (13.4)	30.1 (17.0)	31.9 (18.8)
PGA, n (%):				
Mild	6 (12.0)	8 (16.3)	11 (22.4)	9 (18.8)
Moderate	41 (82.0)	39 (79.6)	33 (67.3)	33 (68.8)
Severe	3 (6.0)	2 (4.1)	5 (10.2)	6 (12.5)
Median cell counts, cells/mm^3^ (Q25, Q75):				
T (CD3^+^)	1310 (982, 1517)	1115 (894, 1455)	1206 (1047, 1578)	1162 (935, 1510)
T_H_ (CD3^+^/CD4^+^)	802 (615, 975)	730 (572, 931)	868 (637, 998)	744 (597, 950)
T_C_ (CD3^+^/CD8^+^)	431 (287, 570)	331 (246, 498)	392 (264, 501)	386 (292, 539)
B (CD19^+^)	195 (134, 304)	241 (136, 322)	198 (163, 300)	247 (143, 343)
NK (CD16^+^/CD56^+^)	159 (93, 188)	135 (91, 214)	130 (95, 207)	152 (97, 216)
Median CMV viral load, copies/500 ng total DNA (Q25, Q75)	0 (0.00, 0.00)	0 (0.00, 0.00)	0 (0.00, 0.00)	0 (0.00, 0.00)
Median EBV viral load, copies/500 ng total DNA (Q25, Q75)	0 (0.00, 1.30)	0 (0.00, 0.95)	0 (0.00, 1.00)	0 (0.00, 0.85)
Median CRP, mg/L (Q25, Q75)	1.84 (0.83, 4.41)	2.54 (1.13, 7.79)	1.92 (1.02, 5.84)	3.14 (0.92, 7.86)

B, B cells; BSA, body surface area; BID, twice daily; CMV, cytomegalovirus; CRP, C-reactive protein; DNA, deoxyribonucleic acid; EBV, Epstein-Barr virus; n, number; NK, natural killer cells; PASI, Psoriasis Area and Severity Index; PGA, Physician’s Global Assessment (scored on a five-point severity scale); Q, quartile; SD, standard deviation; T, total T cells; T_C_, cytotoxic T cells; T_H_, T helper cells.

### Effects on T cells

**Figure 1:**
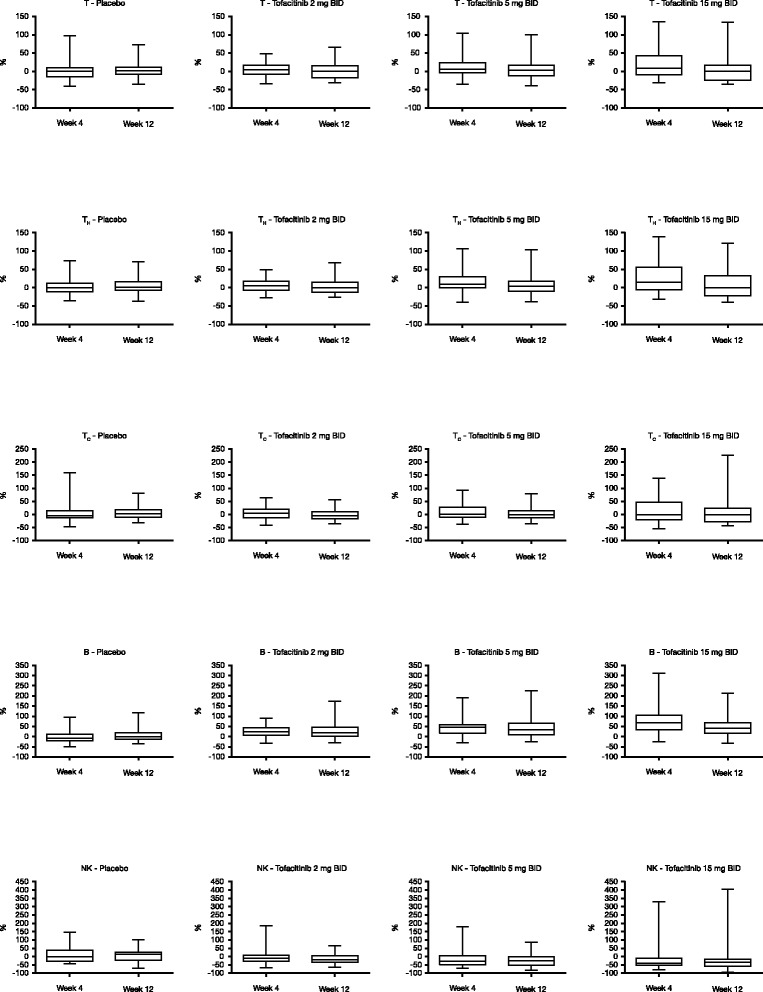
Median percent change from baseline in lymphocyte sub-population cell counts. The horizontal line within each box represents median, with the bottom and top of each box representing the 1^st^ and 3^rd^ quartiles, respectively. Error bars represent minimum and maximum values. B, B cells; BID, twice daily; NK, natural killer cells; T, total T cells; T_H_, T helper cells; T_C_, cytotoxic T cells.

In patients receiving tofacitinib, there was a dose-dependent percentage increase from baseline in median total T (CD3^+^) cell count at Week 4, returning to near baseline levels by Week 12 (Table 2, Figure 1). Such a trend was not observed in patients receiving placebo.

**Table 2:** **Change from baseline in lymphocyte sub-populations, EBV and CMV DNA counts, and CRP** ^**a**^

**Median percent change in lymphocyte sub-population cell counts, cells/mm** ^**3**^ **(Q25, Q75)**					
	**Time point**	**Placebo**	**Tofacitinib**	**Tofacitinib**	**Tofacitinib**
			**2 mg BID**	**5 mg BID**	**15 mg BID**
T (CD3^+^)	Week 4	−0.39 (−14.03, 9.75)	4.79 (−7.45, 17.57)	6.36 (−4.05, 24.24)	8.38 (−9.07, 42.80)
	Week 12	2.28 (−8.31, 11.10)	0.10 (−17.18, 15.87)	3.73 (−11.75, 17.54)	0.65 (−23.86, 17.64)
T_H_ (CD3^+^/CD4^+^)	Week 4	−0.14 (−10.71, 12.20)	5.52 (−6.73, 18.12)	9.56 (0, 30.24)	15.09 (−5.39, 56.12)
	Week 12	0.89 (−7.00, 15.95)	−0.90 (−12.29, 15.42)	3.82 (−9.54, 17.99)	−0.82 (−22.29, 32.57)
T_C_ (CD3^+^/CD8^+^)	Week 4	−4.79 (−12.42, 15.01)	5.13 (−13.33, 19.62)	0.29 (−9.45, 27.35)	−0.23 (−19.05, 47.92)
	Week 12	3.74 (−11.43, 18.42)	−5.58 (−16.27, 11.29)	−1.48 (−12.46, 14.07)	−0.33 (−28.53, 24.61)
B (CD19^+^)	Week 4	−6.87 (−19.05, 11.40)	23.88 (6.60, 43.13)	45.17 (16.28, 56.84)	67.90 (32.64, 103.79)
	Week 12	−0.67 (−12.93, 18.09)	18.03 (0.94, 47.37)	35.32 (9.66, 66.32)	42.86 (16.74, 67.50)
NK (CD16^+^/CD56^+^)	Week 4	0 (−27.68, 37.60)	−10.94 (−28.17, 8.28)	−26.11 (−46.71, 4.35)	−40.80 (−52.50, −10.06)
	Week 12	13.87 (−21.43, 26.67)	−20.00 (−34.17, 3.97)	−22.76 (−51.38, −1.59)	−34.31 (−57.07, −16.57)
**Median change in viral load, copies/500 ng total DNA (Q25, Q75)**					
CMV	Week 4	0 (0.00, 0.00)	0 (0.00, 0.00)	0 (0.00, 0.00)	0 (0.00, 0.00)
	Week 8	0 (0.00, 0.00)	0 (0.00, 0.00)	0 (0.00, 0.00)	0 (0.00, 0.90)
	Week 12	0 (0.00, 0.00)	0 (0.00, 0.00)	0 (0.00, 0.00)	0 (0.00, 0.00)
EBV	Week 12	0 (−1.10, 0.00)	0 (0.00, 1.25)	0 (0.00, 0.60)	0.70 (0.00, 2.90)
**Median percent change in CRP, % (Q25, Q75)**					
CRP	Week 12	9.42 (−52.41, 61.08)	−24.71 (−47.43, 5.49)	−36.31 (−74.62, 5.49)	−69.17 (−82.20, −34.38)

^a^Safety set, no imputation.

B, B cells; BID, twice daily; CMV, cytomegalovirus; CRP, C-reactive protein; DNA, deoxyribonucleic acid; EBV, Epstein-Barr virus; NK, natural killer cells; Q, quartile; T, total T cells; T_H_, T helper cells; T_C_, cytotoxic T cells.

A similar pattern of dose-dependent percentage increases at Week 4, followed by a return to near baseline levels at Week 12, was observed in median T_H_ (CD3^+^/CD4^+^) cell counts (Table 2, Figure 1). No patient had a T_H_ cell count <200 cells/mm^3^. Among patients with a T_H_ cell count of ≥500 cells/mm^3^ at baseline (n = 170), two, two, one, and three patients receiving placebo, tofacitinib 2 mg, 5 mg, or 15 mg BID, respectively, had a single post-baseline measurement of <500 cells/mm^3^. Only one patient had two consecutive T_H_ cell counts <500 cells/mm^3^ (Week 4 and Week 12). This patient received tofacitinib 2 mg BID. While there was some variability in T_C_ (CD3^+^/CD8^+^) cell counts, these changes did not appear to be dose dependent (Table 2, Figure 1).

Overall, there was a good correlation between total lymphocyte count and sub-population cell counts (T cells, T_H_ cells, and T_C_ cells) across groups, ranging from 0.62–0.93 at baseline, 0.58–0.94 at Week 4, and 0.56–0.92 at Week 12.

### Effects on B cells

Tofacitinib treatment resulted in dose-dependent percentage increases from baseline in B cell counts that were sustained throughout the study (Table 2, Figure 1). At Week 12, five (13.2%), two (5.4%), and four (10.3%) patients receiving tofacitinib 2 mg, 5 mg, and 15 mg BID, respectively, had B cell counts above the normal range, compared with one patient (3.0%) in the placebo group.

### Effects on natural killer cells

Treatment with tofacitinib resulted in dose-dependent percentage reductions from baseline in median NK cell count at Week 4. At Week 12 the median NK cell count reduced further in the tofacitinib 2 mg BID group, remained similar in the tofacitinib 5 mg BID group, and increased in the tofacitinib 15 mg BID group (Table 2, Figure 1).

### Changes in C-reactive protein levels

Median CRP values at baseline are shown in Table 1. At Week 12, there were dose-dependent percentage reductions from baseline in median CRP levels in the tofacitinib groups, whereas CRP increased in the placebo group (Table 2).

### Cytomegalovirus and Epstein-Barr virus DNA

**Figure 2:**
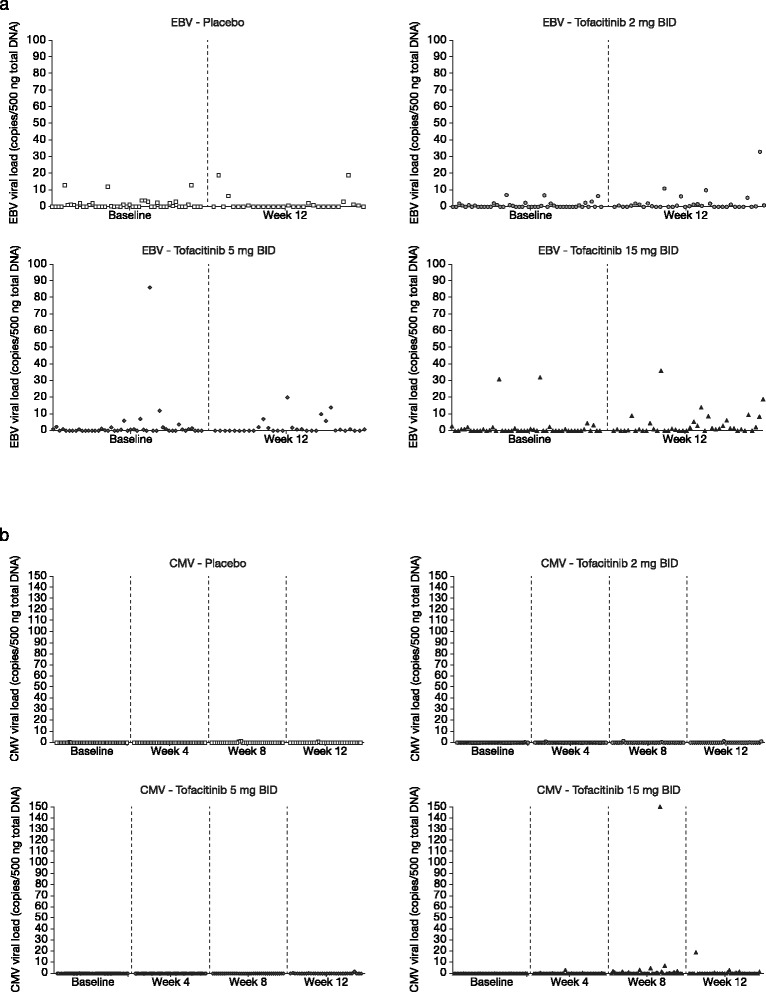
Viral DNA count at baseline and post-baseline in patients receiving tofacitinib or placebo. For: **a)** CMV and **b)** EBV; each data point represents a measurement for an individual patient. BID, twice daily; CMV, cytomegalovirus; EBV, Epstein-Barr virus.

The median (quartile [Q]25, Q75) CMV viral load at baseline was 0 copies/500 ng total DNA (0.0, 0.0) in all groups. The median change from baseline in CMV DNA was 0 copies/500 ng total DNA at Week 4, Week 8, and Week 12 (the Q25 and Q75 were 0.0 for all groups and time points except for the tofacitinib 15 mg BID group at Week 8, in which the Q75 value was 0.9) (Table 2, Figure 2).

CMV DNA was detected in three patients at baseline: one each in the placebo, tofacitinib 2 mg, and tofacitinib 5 mg BID groups. In all three patients, the CMV viral load was <1 copy/500 ng total DNA. Only one patient, a 78-year-old white male receiving tofacitinib 15 mg BID, had post-baseline incidences of CMV DNA >10 copies/500 ng total DNA, which were as follows. This patient had a positive test for CMV on Day 57 (150 copies/500 ng total DNA), which was reported as a mild adverse event. He reported no other adverse signs or symptoms at this time and continued study treatment. CMV levels subsequently reduced to 92 copies/500 ng total DNA at Day 70 while on treatment. Once the initial positive test was relayed to the investigator site, study treatment was discontinued on Day 86, and on Day 87 his CMV level was 19 copies/500 ng total DNA. The event subsequently resolved. Another patient had a single incidence of CMV DNA ≥5 copies/500 ng total DNA. This patient received tofacitinib 15 mg BID, and had a CMV viral load of 7 copies/500 ng total DNA at Week 8, with no adverse events reported. The patient discontinued treatment and the elevated CMV levels resolved.

No comparison of baseline vs post-baseline CMV detectability was possible for patients with detectable CMV at baseline, due to limited sample size (Tables 1 and 3). In patients with undetectable CMV at baseline, tofacitinib was associated with detectable CMV at one or more time points post-baseline (Chi-squared test, p = 0.0001; Table 3). When individual tofacitinib groups were evaluated, the number of patients in whom CMV became detectable post-baseline was significant in the tofacitinib 15 mg BID group only (p = 0.004 vs placebo).

**Table 3:** **Changes from baseline in CMV and EBV viral load in patients receiving placebo or tofacitinib 2 mg, 5 mg or 15 mg BID**

	**Baseline status**	**Post-baseline status**		**Chi-squared p value (vs placebo)**	**Overall p value (vs baseline)**
**Patients with CMV undetectable at baseline**	**Undetectable N (n)**	**Remained undetectable n (%)**	**Became detectable n (%)**		
Placebo	49 (46)	44 (95.7)	2 (4.3)	N/A	0.0001
Tofacitinib 2 mg BID	48 (46)	41 (89.1)	5 (10.9)	0.2381	
Tofacitinib 5 mg BID	48 (45)	42 (93.3)	3 (6.7)	0.6274	
Tofacitinib 15 mg BID	49 (48)	32 (66.7)	16 (33.3)	0.0004	
**Patients with CMV detectable at baseline** ^**a**^	**Detectable N (n)**	**Remained detectable n (%)**	**Became undetectable n (%)**		
Placebo	1 (1)	0 (0)	1 (100.0)	N/A	–
Tofacitinib 2 mg BID	1 (1)	0 (0)	1 (100.0)	–	
Tofacitinib 5 mg BID	1 (1)	0 (0)	1 (100.0)	–	
Tofacitinib 15 mg BID	0 (0)	0 (0)	0 (0)	–	
**Patients with EBV undetectable at baseline**	**Undetectable N (n)**	**Remained undetectable n (%)**	**Became detectable n (%)**		
Placebo	28 (20)	18 (90.0)	2 (10.0)	N/A	0.0122
Tofacitinib 2 mg BID	30 (25)	14 (56.0)	11 (44.0)	0.0124	
Tofacitinib 5 mg BID	26 (19)	15 (78.9)	4 (21.1)	0.3390	
Tofacitinib 15 mg BID	31 (28)	14 (50.0)	14 (50.0)	0.0038	
**Patients with EBV detectable at baseline**	**Detectable N (n)**	**Remained detectable n (%)**	**Became undetectable n (%)**		
Placebo	21 (19)	9 (47.4)	10 (52.6)	N/A	0.1299
Tofacitinib 2 mg BID	18 (13)	9 (69.2)	4 (30.8)	0.2208	
Tofacitinib 5 mg BID	21 (14)	10 (71.4)	4 (28.6)	0.1669	
Tofacitinib 15 mg BID	17 (14)	12 (85.7)	2 (14.3)	0.0236	

^a^A statistical comparison of change in CMV detectability from detectable at baseline to undetectable post-baseline was not possible due to limited sample size.

BID, twice daily; CMV, cytomegalovirus; EBV, Epstein-Barr virus; N, number of patients with baseline values; n, number of patients with baseline and post-baseline values; N/A, not applicable.

The median (Q25, Q75) EBV viral load at baseline was 0.0 copies/500 ng total DNA (0.0, 1.3) in all groups (Table 1). Twelve patients had baseline EBV DNA ≥5 copies/500 ng total DNA: three receiving placebo, and three, four, and two patients receiving tofacitinib 2 mg, 5 mg, and 15 mg BID, respectively. Of these, seven patients (three in the placebo group, and two each in the tofacitinib 5 mg and 15 mg BID groups) had EBV DNA >10 copies/500 ng total DNA. The highest EBV DNA value at baseline (86 copies/500 ng total DNA) was reported for a patient in the tofacitinib 5 mg BID group. This patient had a mild, upper respiratory tract infection at study entry, which began 12 days before treatment and resolved on Day 15.

At Week 12, the median (Q25, Q75) change from baseline in EBV viral load was 0 (–1.1, 1.3) for all groups except for the tofacitinib 15 mg BID group, in which the median EBV DNA count increased by 0.7 copies/500 ng total DNA (0.0, 2.9) (Table 2). Twenty-two patients had EBV DNA ≥5 copies/500 ng total DNA at Week 12: three receiving placebo, and five, five, and nine patients receiving tofacitinib 2 mg, 5 mg, and 15 mg BID, respectively. Of these, nine patients (two each in the placebo, tofacitinib 2 mg, and 5 mg BID groups, and three in the tofacitinib 15 mg BID group) had EBV DNA >10 copies/500 ng total DNA. The highest EBV DNA values at Week 12 were reported for a patient receiving tofacitinib 2 mg BID and a patient receiving tofacitinib 15 mg BID (33 copies and 36 copies/500 ng total DNA, respectively). Both had EBV levels ≥5 copies/500 ng total DNA at baseline. The patient with EBV levels of 33 copies/500 ng total DNA had adverse events of increased low-density lipoprotein and increased blood cholesterol reported at that time point. The patient with EBV DNA 36 copies/500 ng total DNA had no adverse events reported.

Overall, in patients with undetectable EBV at baseline, tofacitinib was associated with detectable EBV at Week 12 (Chi-squared test, p = 0.0122). The number of patients in whom EBV became detectable post-baseline was significant vs placebo in the tofacitinib 2 mg BID (p = 0.0124) and 15 mg BID (p = 0.0038) groups (Table 3). However, there was also a significant proportion of patients in the tofacitinib 15 mg BID group who had detectable EBV at baseline and undetectable EBV at Week 12 (p = 0.0236 vs placebo) (Table 3).

### Correlation analyses

Spearman’s rank correlation coefficient (rho) analyses of pooled patient data showed that EBV viral load, but not CMV viral load, was weakly correlated with larger changes in total T cell, T_H_ cell, T_C_ cell, and B cell counts after 12 weeks of treatment (rho: 0.19, 0.20, 0.17, and 0.25, respectively). Changes in CRP level showed a weak inverse correlation with larger changes in total T cell, T_H_ cell, T_C_ cell, and B cell counts after 12 weeks of treatment (rho: −0.28, −0.26, −0.20, and −0.40, respectively) (Table 4).

**Table 4:** **Spearman’s rank correlation coefficients between change from baseline to week 12 in CMV or EBV viral load, or CRP level, and change in lymphocyte sub-populations**

**Change in outcome variables, rho (p value)**	**Lymphocyte sub-populations**				
	**T (CD3** ^**+**^ **)**	**T**_**H**_ **(CD3** ^**+**^ **/CD4** ^**+**^ **)**	**T**_**C**_ **(CD3** ^**+**^ **/CD8** ^**+**^ **)**	**B (CD19** ^**+**^ **)**	**NK (CD16** ^**+**^ **/CD56** ^**+**^ **)**
CMV viral load, copies/500 ng total DNA	−0.0499 (0.5742)	−0.0889 (0.3164)	0.0020 (0.9817)	−0.0530 (0.5505)	−0.0148 (0.8674)
EBV viral load, copies/500 ng total DNA	0.1872 (0.0351)	0.1970 (0.0264)	0.1711 (0.0544)	0.2491 (0.0047)	0.0033 (0.9709)
CRP level, mg/L	−0.2832 (0.0008)	−0.2644 (0.0017)	−0.2009 (0.0181)	−0.3996 (0.0000)	0.0699 (0.4152)

The correlation analyses presented above were performed using pooled data from the placebo and tofacitinib groups.

B, B cells; CMV, cytomegalovirus; CRP, C-reactive protein; DNA, deoxyribonucleic acid; EBV, Epstein-Barr virus; NK, natural killer cells; rho, Spearman’s rank correlation coefficient; T, total T cells; T_H_, T helper cells; T_C_, cytotoxic T cells.

Potential correlations between tofacitinib dose, lymphocyte sub-population cell counts, CMV and EBV viral load, and CRP level were evaluated. Multivariate analyses using a general linear model detected no correlation between change in CMV or EBV viral load and lymphocyte sub-populations or dose of tofacitinib (Table 5). However, there was strong and significant correlation between change in CRP levels and change in T_C_ cell count (p = 0.043) or dose of tofacitinib (p = 0.026) (Table 5).

**Table 5:** **Correlation analysis results from a general linear model with change from baseline to week 12 in CMV or EBV viral load or change in CRP level as the response variable, and dose of tofacitinib and change in lymphocyte sub-populations as explanatory variables**

**Change in response variables**		**Explanatory variables**					
		**Dose of tofacitinib** ^**a**^	**Lymphocyte sub-populations**				
			**T (CD3** ^**+**^ **)**	**T**_**H**_ **(CD3** ^**+**^ **/CD4** ^**+**^ **)**	**T**_**C**_ **(CD3** ^**+**^ **/CD8** ^**+**^ **)**	**B (CD19** ^**+**^ **)**	**NK (CD16** ^**+**^ **/CD56** ^**+**^ **)**
CMV viral load, copies/500 ng total DNA	df	3	1	1	1	1	1
	F value	1.26	0.14	0.04	0.68	0.00	0.03
	p value	0.2926	0.7047	0.8411	0.4112	0.9925	0.8592
EBV viral load, copies/500 ng total DNA	df	3	1	1	1	1	1
	F value	0.50	0.54	1.69	0.11	0.02	0.57
	p value	0.6807	0.4627	0.1955	0.7430	0.8906	0.4501
CRP level, mg/L	df	3	1	1	1	1	1
	F value	3.18	1.45	1.84	4.18	0.11	1.83
	p value	0.0262	0.2315	0.1775	0.0430	0.7450	0.1784

^a^Doses of tofacitinib considered in this analysis were 0 mg, 2 mg, 5 mg, and 15 mg BID.

B, B cells; BID, twice daily; CMV, cytomegalovirus; CRP, C-reactive protein; df, degrees of freedom; DNA, deoxyribonucleic acid; EBV, Epstein-Barr virus; NK, natural killer cells; T, total T cells; T_H_, T helper cells; T_C_, cytotoxic T cells.

## Discussion

Clinical data describing the impact of immunosuppressive therapies on the overall risk of infection and serious infection are limited in patients with psoriasis compared with rheumatoid arthritis (RA). However, there is emerging evidence to suggest that the infection risk associated with various therapies used in the RA setting may not be replicated in the psoriasis setting [22-24]. Moreover, effects of psoriasis therapies on immunosurveillance may vary according to the mechanism of action for each agent.

In this investigation, there was no clinically significant relationship between changes in CMV or EBV viral load and changes in lymphocyte sub-populations. Minimal and transient effects on T cell counts were identified in patients receiving tofacitinib, which may be related to modulation of cytokines, resultant modulation of adhesion molecules, and consequent trafficking of T cells. This is supported by dose-dependent percent changes from baseline in median total T cell (CD3^+^) count at Week 4, which appeared to normalize across all doses by Week 12. Dose-dependent percentage changes in median B cell and NK cell count were observed with tofacitinib treatment, with B cell count increased from baseline and NK cell count decreased from baseline at Week 12. Overall, changes in lymphocyte sub-populations observed in this study appear consistent with observations in patients receiving tofacitinib for RA [25].

T_H_ cell counts correlated well with total lymphocyte counts over time, suggesting that tofacitinib does not change the T_H_ cell distribution. A T_H_ cell count <200 cells/mm^3^, which is a known risk factor for opportunistic infections in patients with human immunodeficiency virus (HIV) [26] was not observed in any patient in the present study. In addition, excluding those patients with a T_H_ cell count <500 cells/mm^3^ at baseline, fewer than 5% of patients in each group had a post-baseline T_H_ cell count <500 cells/mm^3^. In other settings in which patients may be immunocompromised, including bone marrow transplantation, a significant correlation with the occurrence of opportunistic infection has been observed only in patients with a T_H_ cell count below 115 cells/mm^3^ [27,28]. Based on data collected during this study, there is no need to routinely monitor levels of lymphocyte sub-populations in patients receiving tofacitinib therapy for plaque psoriasis. However, longer-term observation would be needed to confirm these findings.

In the present study, mean baseline CRP levels for each group ranged from 3.10 to 5.47 mg/L, which is above the 3.0 mg/L threshold for high relative risk of cardiovascular disease [29]. Tofacitinib therapy was associated with dose-dependent reductions in CRP at Week 12, reflecting the anti-inflammatory activity of tofacitinib. This is supported by the significant correlation between tofacitinib dose and changes in CRP. Furthermore, the observed, transient increase from baseline in peripheral lymphocytes in patients receiving tofacitinib, and the inverse correlation between changes in CRP level and larger changes in T cell, T_H_ cell, T_C_ cell, and B cell counts after 12 weeks of treatment, is consistent with the recruitment of lymphocytes to sites of inflammation and release of lymphocytes as inflammation resolves.

There is published evidence to suggest an association between active plaque psoriasis and reactivation of CMV, with one study identifying a correlation between psoriasis disease activity in CMV seropositive patients and severity of CMV-antigenemia [30]. In the present study, there was an overall association between tofacitinib and post-baseline detectability of CMV and EBV DNA, and significant changes in CMV and EBV detectability in the tofacitinib 2 mg and 15 mg BID groups (vs placebo). When considered alongside viral load measurements for individual patients, in most cases, changes in CMV and EBV detectability are likely to reflect only subtle differences in viral load. Indeed, a multivariate analysis showed no correlation between viral load and dose of tofacitinib. Considering the important role of T cells and NK cells in the response to viral infection [31,32], this finding implies that the modest changes in lymphocyte sub-populations observed during 12 weeks of tofacitinib therapy did not translate into clinically significant changes in effective immunosurveillance. Moreover, the lack of changes in viral load also suggests that T cell function, critical for control of viral infections [33], is preserved in patients treated with tofacitinib, at least over the course of 12 weeks. This conclusion is supported by the case of one patient with a treatment-emergent adverse event of elevated CMV, whose CMV levels reduced before stopping treatment and who did not develop any clinical symptoms.

In the HIV setting, a CMV viral load of >200 copies/mL predicts the future occurrence of CMV end organ disease [34]. There is no single treatment threshold for CMV in the transplant setting. However, a CMV viral load cut-off of 5000 copies/mL for pre-emptive therapy was described in a recent study investigating CMV reactivation in 161 liver transplant patients [35]. In another study involving 28 liver transplant patients, the median viral load associated with symptomatic CMV disease was 230,000 copies/mL [36]. These levels are substantially in excess of the maximum CMV level reported in any patient in this study.

There are few published studies describing the effects of other therapies for RA or psoriasis on CMV or EBV viral load. A study with 62 patients, aged 2–28 years, receiving biologic (including infliximab and etanercept) or nonbiologic treatment for juvenile RA, Crohn’s disease, or ulcerative rectocolitis detected CMV DNA in 1.6% of patients and EBV in 4.8% of patients [37]. All but one patient with detectable CMV or EBV had received nonbiologic therapy in the preceding year. Neither CMV nor EBV DNA was detected in the control group, which comprised 62 healthy volunteers. However, the extent to which viral load was related to the underlying disease or the treatment received was unclear [37]. A study of EBV viral load in 122 patients receiving biologic agents, including TNFi, for inflammatory arthritis found that 90% of patients receiving active treatment (and 98% of biologic-naïve controls) had a positive antibody test for EBV during treatment, although subsequent PCR testing revealed no evidence of viral activation [38]. In addition, Niemann and colleagues detected no EBV DNA in 26 patients with psoriasis who received at least eight weeks of treatment with various nonbiologic and biologic agents (methotrexate, cyclosporine, etanercept, or alefacept) [39]. It should be noted that our investigation, and the above studies by McKeown and Niemann et al., measured viral load in patients who were asymptomatic for infection with CMV or EBV. In other indications where immunosuppressive therapy is used, such as RA and transplantation, an associated risk of viral infections has been observed [40], although the duration of treatment in that study was substantially longer than the 12-week duration of tofacitinib therapy in this study. Thus, the available evidence suggests that, while CMV or EBV DNA may become detectable in some patients during treatment with biologic or nonbiologic therapy for inflammatory diseases such as plaque psoriasis, viral reactivation and symptomatic clinical infections are rare.

Potential limitations of this analysis were the duration of exposure to tofacitinib (12 weeks) and the use of lymphocyte subsets (CD4 and CD8) as surrogates of virus-specific CD4+ and CD8+ cells. While no effect of tofacitinib exposure on viral load was observed over the 12-week study period, one cannot be certain that such an effect would not be observed over a more prolonged period of exposure. With regard to lymphocyte subsets it is possible, albeit unlikely, that tofacitinib treatment could affect the number or function of virus-specific cells without markedly affecting the total number of cells. The absence of an observed increase in viral load suggests that the number and function of these virus-specific cells was sufficient to control viral replication.

## Conclusions

In patients with psoriasis, 12 weeks of tofacitinib treatment had no clinically significant effects on CMV or EBV viral load, suggesting that lymphocyte sub-populations critical to the response to chronic viral infections and viral reactivation were not significantly affected. Replication of these findings during long-term use of tofacitinib will allow confirmation of this observation.

## Abbreviations

ANCOVA: Analysis of covariance
B: B cells
BID: Twice daily
BSA: Body surface area
CMV: Cytomegalovirus
CRP: C-reactive protein
df: Degrees of freedom
DNA: Deoxyribonucleic acid
EBV: Epstein-Barr virus
HIV: Human immunodeficiency virus
IL: Interleukin
JAK: Janus kinase
N/A: Not applicable
NK: Natural killer cell
PASI: Psoriasis Area and Severity Index
PGA: Physician’s Global Assessment
Q: Quartile
RA: Rheumatoid arthritis
rho: Spearman’s rank correlation coefficient
SD: Standard deviation
T: Total T cells
T_C_: Cytotoxic T cell
T_H_: T helper cell
TNFi: Tumor necrosis factor inhibitor
